# A collection of Australian *Drosophila* datasets on climate adaptation and species distributions

**DOI:** 10.1038/sdata.2015.67

**Published:** 2015-11-24

**Authors:** Sandra B. Hangartner, Ary A. Hoffmann, Ailie Smith, Philippa C. Griffin

**Affiliations:** 1 School of BioSciences, The University of Melbourne, Parkville, Victoria 3010, Australia; 2 School of Biological Sciences, Monash University, Clayton, Victoria 3800, Australia; 3 eScholarship Research Centre, The University of Melbourne, Parkville, Victoria 3010, Australia

**Keywords:** Experimental evolution, Population genetics, Climate-change ecology, Evolutionary biology, Evolutionary genetics

## Abstract

The Australian *Drosophila* Ecology and Evolution Resource (ADEER) collates Australian datasets on drosophilid flies, which are aimed at investigating questions around climate adaptation, species distribution limits and population genetics. Australian drosophilid species are diverse in climatic tolerance, geographic distribution and behaviour. Many species are restricted to the tropics, a few are temperate specialists, and some have broad distributions across climatic regions. Whereas some species show adaptability to climate changes through genetic and plastic changes, other species have limited adaptive capacity. This knowledge has been used to identify traits and genetic polymorphisms involved in climate change adaptation and build predictive models of responses to climate change. ADEER brings together 103 datasets from 39 studies published between 1982–2013 in a single online resource. All datasets can be downloaded freely in full, along with maps and other visualisations. These historical datasets are preserved for future studies, which will be especially useful for assessing climate-related changes over time.

## Background & Summary

The Australian *Drosophila* Ecology and Evolution Resource (ADEER) contains three main *Drosophila* data collections: (1) clinal data, (2) species distribution data and (3) genomics data. The clinal and species distribution collections are described in this data descriptor, whereas the genomics data will be described elsewhere. The majority of data was generated by Ary Hoffmann’s research group at the University of Melbourne, with contributions from several other Australian researchers (see Acknowledgements).

*Drosophila* species have long been used as model organisms to answer fundamental questions in biology, and the most intensively studied *Drosophila* species (in particular *Drosophila melanogaster*) are Northern Hemisphere in origin^1^. Drosophilids as a broader taxonomic group are very diverse in Australia, with over 300 species identified in the tropical and temperate forests located on the east coast. Australia contains a disproportionately large number of species in the genus *Scaptodrosophila*, many of them endemic to this continent^2^. The ADEER collection broadens the scope of worldwide drosophilid data, by focussing on clinal patterns in traits and genes in Australian drosophilids as well as on thoroughly-studied species distributions. This collection contains data on the ecology and evolution of eleven species in the genera *Drosophila* and *Scaptodrosophila* including rainforest specialists (e.g. *D. birchii*), endemic species (e.g. *D. bunnanda*) and cosmopolitan species (e.g. *D. melanogaster*).

The east coast of Australia spans a gradient of climatic conditions from cool-temperate Tasmania to tropical northern Queensland. This gradient is unique as it occurs within a narrow elevation range and on a small continent with an ancient geology and a rich biodiversity with a high proportion of endemic species across several biomes^3^. The gradient provides a model system for studying equivalent climatic gradients on other continents, and represents an outstanding natural laboratory for the study of traits and genes that are associated with climatic adaptation^4^. Changes in traits and genes along this gradient (i.e. clines) can arise by natural selection, producing continuous patterns over geographic space. The eastern Australian gradient has been used to investigate the involvement of numerous phenotypic traits and genetic markers in climate adaptation^4^. The clinal data collection contains data from eight species from studies published between 1982–2013 and includes morphological, life-history, stress resistance traits as well as genetic markers. Most of these studies used common garden experiments to test for clinal variation, but some studies were performed in the field.

The species distribution collection includes five species from the *melanogaster* species group (*montium* subgroup) and two species from the *repleta* species group within the genus *Drosophila*, as well as two *Scaptodrosophila* species. These datasets contain presence records from field collections between 1924 and 2013 which are based on previously-published records in the literature, collections made by the dataset authors, and specimens in the Australian Museum^5–7^. Many species are restricted to the tropics, a few are temperate specialists, and some are broadly distributed across climatic regions. The varied distributions of drosophilid species along the temperate–tropical cline provide a powerful tool for studying climate adaptation and species distribution limits.

Previous work on *Drosophila* species using the Australian cline has demonstrated that monitoring biological changes along geographic climate gradients is a powerful approach for detecting evolutionary shifts under climate change^8,9^. Ongoing data collections from the eastern Australia cline provide an opportunity to monitor phenotypic traits and genetic markers by comparison to historical data, as climate change proceeds. Such temporal studies are particularly useful for tracking continuing evolutionary responses to climate change as well as dynamically projecting species distributions under ongoing climate change scenarios.

## Methods

### Clinal data collection

**Figure 1:**
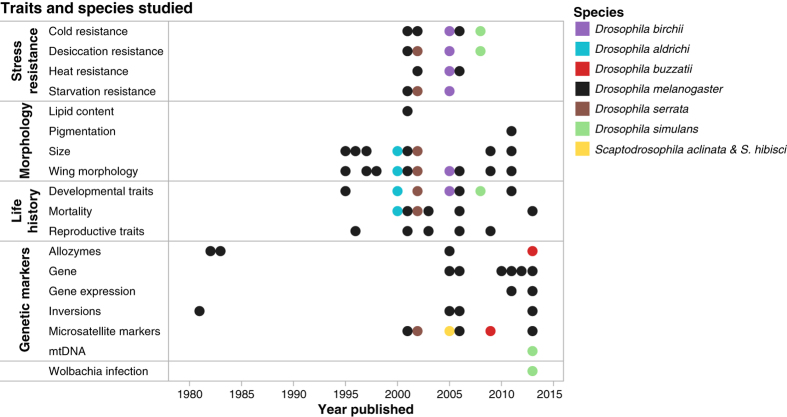
An overview of the trait groups and species studies in the datasets of ADEER. All datasets were published between 1982 and 2013 in a total of 39 papers.

The clinal data collection contains data on morphological, life-history and stress resistance traits, as well as genetic marker data (Tables 1 and 2 (available online only), Fig. 1). All datasets of this kind involve flies collected at multiple locations within their geographic distribution, usually along a north-south gradient on the east coast of Australia. Material and methods for each dataset appear in detail in the original publication; here we provide a general summary of the approaches used.

**Figure 2:**
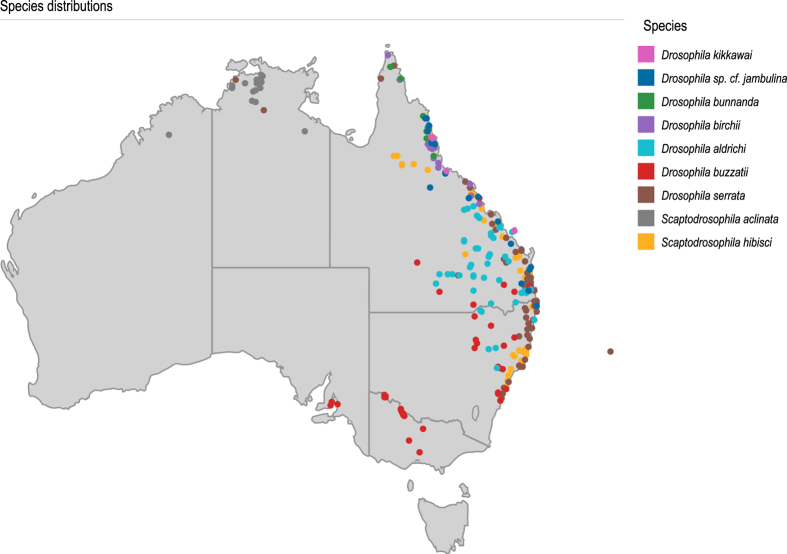
Collection records are shown for nine drosophilid species (*D. aldrichi*, *D. birchii*, *D*. *bunnanda*, *D. buzzatii*, *D. kikkawai*, *D. serrata*, *D.* sp. cf. *jambulina*, *S. aclinata* and *S. hibisci*). These data were collected between 1924 and 2005 and are based on records in the literature, collections made by the dataset authors and specimens in the Australian Museum.

This clinal data collection includes data recorded at the level of the individual fly (46 datasets), the subgroup level (12 datasets) or the population level (41 datasets). The term population is here used for a group of flies collected at a single geographic location. Distinct collection sites were typically at least 40 km apart. Individual data include morphological, life-history, stress resistance traits and individual genotype at genetic marker loci. Many data were recorded as population frequencies, such as *Wolbachia* infection rate and genetic marker frequency (Table 1 (available online only) and Tables 2 (available online only) and 3). For other datasets the data are available as population means, including morphological, life-history and stress resistance traits and genetic markers. A few datasets report results at the subgroup level. These datasets include traits that were measured per vial (e.g. development time, longevity and mortality), per cage (e.g. mortality and fecundity) or per group of flies (desiccation and starvation resistance, gene expression). In addition, one dataset reports data on isofemale lines^10^. Isofemale lines are fly lines that were founded from the offspring of one single wild female (Figs 1 and 2).

Fly populations compared for clinal variation in quantitative traits have almost always been maintained in the laboratory prior to testing, for periods ranging from just one generation to several years. The effect of laboratory culture on clinal patterns was specifically investigated in two of the datasets included in this collection using *D. melanogaster* ^11,12^. Almost all clinal studies on quantitative traits in this collection involve a common garden design, where populations are reared in a common environment before they are tested for a specific trait. Flies are therefore kept under controlled temperature and day length and on standard fly medium within a study, but these conditions can vary substantially among studies. A few studies did not use common garden experiments. These include the two studies mentioned above^11,12^ where field flies were preserved in alcohol to measure wing traits. Other exceptions^13,14^ involved clinal variation in fecundity and mortality scored directly under field conditions.

### Morphological traits

27 datasets from 15 publications in this collection investigated morphological traits in *D. melanogaster*, *D. serrata*, *D. aldrichi*, *D. buzzatii*, *D. simulans*, or *D. birchii.* The morphological traits include size (10 datasets), wing morphology (20 datasets), pigmentation (2 datasets) and lipid content (1 dataset).

Thorax length, measured from the anterior margin of the thorax to the posterior tip of the scutellum, is most often used as a measure of size and was investigated in four *D. melanogaster* studies^10,15–17^ in *D. aldrichi* and *D. buzzatii*^18^ and in *D. simulans*^16^. Mass was used as a measure of size in one *D. serrata* study^19^. In addition, egg size was measured in one *D. melanogaster* study^20^.

Wing morphology was investigated in seven *D. melanogaster* studies^11,12,15–17,21,22^, in *D. aldrichi* and *D. buzzatii*^18^, in *D. birchii*^23^, in *D. serrata*^19,24^ and in *D. simulans*^16^. Wings were removed from individual flies and mounted on slides, and wing traits were either directly measured under a microscope^11,12,17,22^ or measured from landmarked images captured under the microscope^15,16,18,19,21^.

Pigmentation was investigated in one *D. melanogaster* study and was scored by visual examination using four phenotypic classes^25^.

Lipid levels were scored in one *D. melanogaster* study where adult females were initially dried in an oven for 48 h and then soaked in ether for 24 h to extract the lipids^9^.

### Life-history traits

There are 24 datasets in this collection from 13 publications that investigated life-history traits in *D. melanogaster*, *D. serrata*, *D. aldrichi*, *D. buzzatii*, *D. simulans*, or *D. birchii.* This includes traits related to development (11 datasets), mortality (8 datasets) and reproduction (8 datasets).

Egg-to-adult development time was investigated in two *D. melanogaster* studies^21,26^, *D. aldrichi* and *D. buzzatii*^18^, *D. birchii*^23^, *D. serrata*^27^ and in *D. simulans*^28^. Development time was measured from the midpoint of the egg laying period to adult eclosion (emergence from the pupal case). In addition, egg development stage was examined in female *D. melanogaster* after being exposed to diapause-inducing conditions for 28 days^29^.

Mortality was investigated in *D. melanogaster*^13,14,30^, *D. aldrichi* and *D. buzzatii*^18^ and *D. serrata*^27^. In *D. melanogaster*, mortality was recorded in field cages held at temperate winter conditions near Melbourne^13^ and at tropical winter conditions in Cairns^14^. In addition, longevity of once-mated females was scored under standard laboratory conditions^30^. The flies were transferred to fresh vials every day, and at each transfer, all vials were examined for dead flies^30^. In *D. aldrichi* and *D. buzzatii*, larvae to adult viability was scored after rearing the flies at three temperatures treatments^18^. Egg to adult and pupal to adult viability were scored in *D. serrata* collected before and after winter^27^. To score egg to adult and pupae to adult viability, vials were scored until no new adults emerged and the number of pupae in each vial was counted to obtain pupal viability data^27^.

Reproductive traits were investigated in five *D. melanogaster* studies^13–15,20,21^. Overwintering fecundity was recorded in field cages held at temperate winter conditions near Melbourne^13^ and at tropical winter conditions in Cairns^14^. Rako *et al.*^15^ tested for the maintenance of fertility in males that have survived in field cages held at temperate winter conditions near Melbourne. Males were crossed to virgin females and the number of offspring was scored for each male^15^. Ovariole number was scored in two studies, whereas the number of ovarioles in each ovary was counted directly after dissection of the females^20,21^.

### Stress resistance traits

Sixteen datasets from 8 publications investigated stress traits in *D. melanogaster*, *D. serrata*, *D. simulans*, or *D. birchii.* These traits include cold resistance (8 datasets), desiccation resistance (6 datasets), heat resistance (4 datasets) and starvation resistance (5 datasets).

Cold resistance, scored as chill coma recovery time was investigated in *D. melanogaster*^21,31^, *D. birchii* ^23^, *D. serrata*^19^ and *D. simulans*^28^. Flies were placed in empty vials which were immersed in a 10% glycol solution cooled to a constant temperature of 0 °C . After 1–8 h, vials were removed from the cold bath and placed at room temperature and recovery time of flies was scored^19,21,23,28,31^. Cold resistance scored as mortality after chill coma was investigated in *D. melanogaster*^10,31^ and *D. serrata*^27^. Groups of females were placed into empty vials and submerged in a −2 °C waterbath for 1–3 h. Flies were allowed to recover in vials with fly medium for 24–48 h before scoring mortality^10,27,31^.

Desiccation resistance was investigated in *D. melanogaster*^10,32^, *D. serrata*^19^, *D. simulans*^28^ and *D. birchii*^23^. Flies were placed in empty vials covered with gauze and then transferred to a desiccator with silica gel left at 25 °C . Mortality was scored hourly until all flies had died^10,19,23,28,32^.

Heat resistance was investigated in *D. melanogaster*^21,31,32^, and *D. birchii*^23^. Individual flies were placed into 5 ml glass vials submerged into a glass tank with water held at 39 °C (38.5 °C for *D. birchii*). Resistance was scored as the time taken for flies to be knocked down^21,23,31,32^.

Starvation resistance was investigated in *D. melanogaster*^10,32^, *D. birchii*^23^ and *D. serrata*^19^. Flies were placed in vials/tubes containing agar and these vials were placed in a chamber with water to maintain humidity close to 100%. Chambers were held at 25 °C and mortality was scored at 6–8 h intervals until at least half the flies had died^10,19,32^. Griffiths *et al.*^23^ scored starvation resistance by placing flies in vials, which were then inverted over a second vial containing cotton wool and water. Flies in the vial were separated from the water with fine gauze and the two vials were sealed together with Parafilm®. The flies were scored for survival every hour until half the flies had died^23^.

### Genetic markers

This data collection contains 41 datasets from 20 publications that investigated genetic markers in *D. melanogaster*, *D. serrata*, *D. buzzatii*, *D. simulans*, *S. aclinata* or *S. hibisci.* Genetic marker types include allozymes (9 datasets), DNA sequence polymorphism (18 datasets), DNA repeat variation (i.e. microsatellites, 8 datasets), gene expression levels (4 datasets), inversion polymorphisms (5 datasets), and mitochondrial DNA regions (1 dataset).

Allozymes are enzymes that differ in electrophoretic mobility as a result of allelic differences at a single locus^33^. Allozymes were investigated in *D. melanogaster*^8,34–36^ and *D. buzzatii*^37^. Allozymes were scored after electrophoresis of single fly homogenates and staining^8,34–37^. Adh and Pgd were scored in *D. melanogaster*^8,34–36^ and *D. buzzatii*^37^. Gpdh, G6pd and Pgd were scored in *D. melanogaster*^35,36^ and Aldox, Hex, Est1, Est2 and Lap were scored in *D. buzzatii*^37^.

DNA sequence polymorphism can be determined using polymerase chain reaction (PCR) followed by gel electrophoresis (to detect size variation) or sequencing (to detect sequence variation)^33^. *Drosophila melanogaster* has been intensively used as a model to study DNA sequence polymorphisms along the eastern Australian cline^21,29,30,38–42^. In addition, variation in mitochondrial DNA sequences was investigated in *D. simulans*^43^. Several genes have been investigated in *D. melanogaster*: *clock* and *period*^40^, *couch potato*^29^, *drosophila cold acclimation*^39^, *frost*^38^, *hsp70* (ref. 21), *hsr-omega*^21,41^, *methuselah*^30^, and *neurofibromin*^42^. The protocols to test for clinal variation in DNA sequence polymorphism varied substantially among the studies. In short, fly DNA was most often extracted using a Chelex/Proteinase K method^42^ but sometimes used a modified CTAB method^40^. Amplification of nuclear and mitochondrial DNA was performed using standard PCR methods and variation in DNA sequences was determined by gel electrophoresis or sequencing. For further details see^21,29,30,38–43^.

Microsatellites are tandemly repeated sequences of 1–6 nucleotides. Microsatellite markers are highly polymorphic and are assumed to evolve neutrally^33^. Microsatellites were investigated in *D. melanogaster*^22,34,41^, *D. buzzatii*^44^, *D. serrata*^27^, *S. aclinata* and *S. hibsici*^5^. After DNA extraction, microsatellite markers were amplified by polymerase chain reaction (PCR) using the unique sequences of flanking regions as primers and then repeat length was measured either by separating radiolabelled products on a gel or separating fluorescent-labelled products on a DNA sequencer. For further details see^5,22,27,34,41,44^.

Gene expression assays aim to quantify the level of RNA transcript present in the cell for each gene of interest using real-time PCR or deep-sequencing technologies^45^. Expression of three genes was investigated in *D. melanogaster*: *couch potato*^29^, *ebony*^25^, and *methuselah*^30^. In each case, RNA was isolated and purified to ensure DNA removal; cDNA was then synthesised for use as template for real-time PCR on the Light-Cycler® 480 (Roche) system and normalized using housekeeping genes. Further details are available in publications^25,29,30^.

Inversion polymorphism refers to the phenomenon of a chromosome region appearing in either standard or ‘reversed’ orientation in a population, which results in multiple genes being inherited together rather than assorting independently. It has been intensively investigated in *D. melanogaster* in Australia. The inversion *In(3R)Payne* is the most frequently studied inversion^8,41,42,46^, but *In(2R)NS*, *In(3L)Payne*, *In(3R)C*^46^ and *In(2L)t*^34,46^ have also been investigated. Two different approaches were used to test for inversion polymorphism: The BI-PASA method genotypes a SNP polymorphism shown to be in complete linkage disequilibrium with *In(3R)Payne* in Australia^8,41,42^. Alternatively, a salivary gland preparation was made from a single 3rd-instar larva and lacto-acetic orcein was used to stain the chromosome. After staining, glands were squashed under a cover slip and visualized with a light microscope to examine banding patterns and loops characteristic of inversion status^34,46^.

#### *Wolbachia*

*Wolbachia* are maternally inherited intracellular bacteria that can manipulate host reproduction^43^. One study in this collection investigated *Wolbachia* infections in *D. simulans*^43^. DNA was extracted using a standard Chelex based method and assays for *Wolbachia* infection status and strain type were performed with a fluorescence-based PCR assays using the Roche LightCycler® 480 system^47^.

### Species distribution collection

The species distribution collection contains data from two *Scaptodrosophila* species and seven *Drosophila* species (Table 1 (available online only), Table 3 and Fig. 2). Schiffer and McEvey^7^ investigated distributions of members of the *montium* subgroup (*Drosophila bunnanda*, *D. serrata*, *D. birchii*, *D. kikkawai* and *D.* sp. cf. *jambulina*) along the east coast of Australia. Collection records are available for 122 locations that were sampled between 1924 and 2005 and data are based on records in the literature, collections made by the authors and specimens in the Australian Museum^7^. Collection records are also available for the cactophilic *D. aldrichi* and *D. buzzatii*^6^. These species were sampled between 1971 and 2002 in 97 locations where *Opuntia* cacti occur and the *Opuntia* species were recorded for each location. Barker^5^ collected distribution data of *S. aclinata* and *S. hibisci* which are both restricted to *Hibiscus* flowers^5^. *Scaptodrosophila aclinata* were sampled in 24 locations in 1995 and *S. hibisci* were sampled in 63 locations in 1998 and the *Hibiscus* species were recorded for all locations. For further details see the relevant publications^5–7^.

## Data Records

All 103 datasets are freely available through the ADEER website (http://adeer.pearg.com/), where additional datasets will be added in the future. In addition to the datasets, ADEER also provides a short description and a visualisation of each dataset and a link to the publication describing the datasets (Data Citation 1). The datasets can be accessed by browsing the collections, species or traits or by using the “Search” function. All 103 datasets are listed under “Browse Datasets” or as a default using the “Search” function. The data can be downloaded by clicking on the “Data Online” icon. A static version of all datasets was also transferred to Dryad on 19.7.2015 (Data citation 2). The datasets 63–70 from the Lee et al. (2011) publication are also freely available on the Dryad repository (Data Citation 3). In addition, the dataset 25 from the Lee et al. (2013) publication (Data Citation 4), the dataset 24 from the Kriesner et al. 2013 publication (Data Citation 5), the datasets 74-76 from the Sgrò et al. (2013) publication (Data Citation 6) and the datasets 37–39 from the Telonis-Scott *et al*. (2011) publication (Data Citation 7) are already freely available on the Dryad repository.

## Technical Validation

All datasets of this collection have been published in peer-reviewed journals confirming the technical quality of the data and the appropriate use of experimental designs. Experimental designs always included control treatments where necessary and careful replication and randomization of the experimental units. All data have also been statistically analysed, which included testing for measurement and recording errors. Furthermore, in the process of collecting this resource, each dataset was visualized and checked for potential inconsistencies. Spelling mistakes were corrected in the datasets, but only datasets where no inconsistencies were found in the data were included in this resource.

## Usage Notes

The annual average daily mean temperature of Australia has risen by 0.9 °C since 1910 (CSIRO 2014) and Australian temperatures are projected to continue to increase by about 2–4 °C by 2100 following the global trend^48^. The increase in average and extreme temperatures presents a major challenge to biodiversity^49^.

The clinal and species distribution datasets will be valuable for temporal comparisons in the future to understand current and future evolutionary responses to climate change and to predict species distributions under ongoing climate change scenarios. Clinal data of phenotypic traits and genetic markers as well as species distributions can be tracked over time and tested for adaptive responses under climate change^8^. In addition, researchers can use the datasets for comparing shifts in species distributions and linking these to climatic variables.

There is now ample evidence that natural populations are responding to climate change by shifting their geographic distribution and phenology^50–52^ and an increasing number of studies have demonstrated evidence for rapid adaptive evolution in response to climate change^53,54^. Although plastic and genetic responses may allow some species to cope with climate change, extinction risks are predicted to be high, in particular in Australia^55^. One major challenge is to identify the most vulnerable species that will not be able to adapt fast enough to keep pace with climate change^52,53^. Collections like this one that span multiple related species with different degrees of adaptive potential and climate tolerance are important for understanding why some species are more vulnerable than others. Once this is better understood in model groups like drosophilid flies, researchers can apply general patterns to mammals, birds, plants and other groups to help prioritise conservation efforts.

## Additional Information

**How to cite this article**: Hangartner, S. B. *et al.* A collection of Australian *Drosophila* datasets on climate adaptation and species distributions. Sci. Data 2:150067 doi: 10.1038/sdata.2015.67 (2015).

## Supplementary Material



## Figures and Tables

**Table 1:** Overview of all datasets included in ADEER

**Data file name**	**Collection**	***Species***	**Trait**	**Trait group**	**Data type**	**Publication**	**DOI / PMID for original publication**	**Data record accession on ADEER**	**DOI for ADEER**	**Data repository**	**DOI for Dryad**
1 Azevedo et al. ^20^ Egg size & ovariole number	Clinal	*Drosophila melanogaster*	Ovariole number and egg size	Life history and morphology	Population means	Azevedo et al. ^20^	10.2307/2410702	http://adeer.pearg.com/biogs/DR00273b.htm	https://dx.doi.org/10.4225/49/555C0B8D30C3E	Dryad	http://dx.doi:10.5061/dryad.k9c31
2 Azevedo et al. ^11^ Wing traits	Clinal	*Drosophila melanogaster*	Wing to aspect ratio	Morphology	Population means	Azevedo et al. ^11^	10.2307/2410702	http://adeer.pearg.com/biogs/DR00277b.htm	https://dx.doi.org/10.4225/49/555C0B8D30C3E	Dryad	http://dx.doi:10.5061/dryad.k9c31
3 Gockel et al. Microsatellite markers	Clinal	*Drosophila melanogaster*	Microsatellite markers	Genetic markers	Individual categories	Gockel et al. ^22^	11333239	http://adeer.pearg.com/biogs/DR00279b.htm	https://dx.doi.org/10.4225/49/555C0B8D30C3E	Dryad	http://dx.doi:10.5061/dryad.k9c31
4 Gockel et al. ^22^ Wing area	Clinal	*Drosophila melanogaster*	Wing area	Morphology	Population means	Gockel et al. ^22^	11333239	http://adeer.pearg.com/biogs/DR00280b.htm	https://dx.doi.org/10.4225/49/555C0B8D30C3E	Dryad	http://dx.doi:10.5061/dryad.k9c31
5 Griffiths et al. ^23^ Development time	Clinal	*Drosophila birchii*	Development time	Life history	Population means	Griffiths et al. ^23^	10.1111/j.1420-9101.2004.00782.x	http://adeer.pearg.com/biogs/DR00283b.htm	https://dx.doi.org/10.4225/49/555C0B8D30C3E	Dryad	http://dx.doi:10.5061/dryad.k9c31
6 Griffiths et al. ^23^ Stress resistance & wing size	Clinal	*Drosophila birchii*	Cold-, desiccation-, heat-, starvation resistance and wing centroid size	Stress resistance and morphology	Population means	Griffiths et al. ^23^	10.1111/j.1420-9101.2004.00782.x	http://adeer.pearg.com/biogs/DR00282b.htm	https://dx.doi.org/10.4225/49/555C0B8D30C3E	Dryad	http://dx.doi:10.5061/dryad.k9c31
7 Hallas et al. ^19^ Stress resistance and size	Clinal	*Drosophila serrata*	Cold-, desiccation- and starvation resistance, mass and wing length	Stress resistance and morphology	Population means	Hallas et al. ^19^	10.1017/S0016672301005523	http://adeer.pearg.com/biogs/DR00315b.htm	https://dx.doi.org/10.4225/49/555C0B8D30C3E	Dryad	http://dx.doi:10.5061/dryad.k9c31
8 Hoffmann & Shirriffs ^24^ Wing traits	Clinal	*Drosophila serrata*	Wing landmarks	Morphology	Individual measurements	Hoffmann & Shirriffs ^24^	10.1111/j.0014-3820.2002.tb01418.x	http://adeer.pearg.com/biogs/DR00257b.htm	https://dx.doi.org/10.4225/49/555C0B8D30C3E	Dryad	http://dx.doi:10.5061/dryad.k9c31
9 Hoffmann et al. ^10^ Desiccation resistance	Clinal	*Drosophila melanogaster*	Desiccation resistance	Stress resistance	Individual measurements	Hoffmann et al. ^10^	10.1111/j.0014-3820.2001.tb00681.x	http://adeer.pearg.com/biogs/DR00259b.htm	https://dx.doi.org/10.4225/49/555C0B8D30C3E	Dryad	http://dx.doi:10.5061/dryad.k9c31
10 Hoffmann et al. ^10^ Starvation resistance	Clinal	*Drosophila melanogaster*	Starvation resistance	Stress resistance	Individual measurements	Hoffmann et al. ^10^	10.1111/j.0014-3820.2001.tb00681.x	http://adeer.pearg.com/biogs/DR00261b.htm	https://dx.doi.org/10.4225/49/555C0B8D30C3E	Dryad	http://dx.doi:10.5061/dryad.k9c31
11 Hoffmann et al. ^10^ Line means	Clinal	*Drosophila melanogaster*	Cold resistance, desiccation resistance, starvation resistance, lipid content and thorax length	Stress resistance and morphology	Subgroup means	Hoffmann et al. ^10^	10.1111/j.0014-3820.2001.tb00681.x	http://adeer.pearg.com/biogs/DR00260b.htm	https://dx.doi.org/10.4225/49/555C0B8D30C3E	Dryad	http://dx.doi:10.5061/dryad.k9c31
12 Hoffmann et al. ^31^ Cold recovery time	Clinal	*Drosophila melanogaster*	Cold recovery time	Stress resistance	Individual measurements	Hoffmann et al. ^31^	10.1046/j.1461-0248.2002.00367.x	http://adeer.pearg.com/biogs/DR00223b.htm	https://dx.doi.org/10.4225/49/555C0B8D30C3E	Dryad	http://dx.doi:10.5061/dryad.k9c31
13 Hoffmann et al. ^31^ Cold resistance survival	Clinal	*Drosophila melanogaster*	Cold resistance survival	Stress resistance	Individual measurements	Hoffmann et al. ^31^	10.1046/j.1461-0248.2002.00367.x	http://adeer.pearg.com/biogs/DR00224b.htm	https://dx.doi.org/10.4225/49/555C0B8D30C3E	Dryad	http://dx.doi:10.5061/dryad.k9c31
14 Hoffmann et al. ^31^ Heat knockdown time	Clinal	*Drosophila melanogaster*	Heat knockdown time	Stress resistance	Individual measurements	Hoffmann et al. ^31^	10.1046/j.1461-0248.2002.00367.x	http://adeer.pearg.com/biogs/DR00225b.htm	https://dx.doi.org/10.4225/49/555C0B8D30C3E	Dryad	http://dx.doi:10.5061/dryad.k9c31
15 Hoffmann et al. ^14^ Overwinter fecundity	Clinal	*Drosophila melanogaster*	Overwinter mortality, overwinter fecundity	Life history	Population means	Hoffmann et al. ^14^	10.1046/j.1420-9101.2003.00561.x	http://adeer.pearg.com/biogs/DR00263b.htm	https://dx.doi.org/10.4225/49/555C0B8D30C3E	Dryad	http://dx.doi:10.5061/dryad.k9c31
16 Hoffmann et al. ^38^ Frost locusA	Clinal	*Drosophila melanogaster*	Frost locus	Genetic markers	Population frequencies	Hoffmann et al. ^38^	10.1111/j.1365-2583.2012.01149.x	http://adeer.pearg.com/biogs/DR00265b.htm	https://dx.doi.org/10.4225/49/555C0B8D30C3E	Dryad	http://dx.doi:10.5061/dryad.k9c31
17 Hoffmann et al. ^38^ Frost locusB	Clinal	*Drosophila melanogaster*	Frost locus	Genetic markers	Population frequencies	Hoffmann et al. ^38^	10.1111/j.1365-2583.2012.01149.x	http://adeer.pearg.com/biogs/DR00266b.htm	https://dx.doi.org/10.4225/49/555C0B8D30C3E	Dryad	http://dx.doi:10.5061/dryad.k9c31
18 James & Partridge ^26^ Development time	Clinal	*Drosophila melanogaster*	Development time	Life history	Population means	James & Partridge ^26^	10.1046/j.1420-9101.1995.8030315.x	http://adeer.pearg.com/biogs/DR00286b.htm	https://dx.doi.org/10.4225/49/555C0B8D30C3E	Dryad	http://dx.doi:10.5061/dryad.k9c31
19 James & Partridge ^26^ Time to pupation	Clinal	*Drosophila melanogaster*	Time to pupation	Life history	Population means	James & Partridge ^26^	10.1046/j.1420-9101.1995.8030315.x	http://adeer.pearg.com/biogs/DR00285b.htm	https://dx.doi.org/10.4225/49/555C0B8D30C3E	Dryad	http://dx.doi:10.5061/dryad.k9c31
20 James et al. ^17^ Thorax length & wing traits	Clinal	*Drosophila melanogaster*	Thorax length and wing traits	Morphology	Population means	James et al. ^17^	7498744	http://adeer.pearg.com/biogs/DR00288b.htm	https://dx.doi.org/10.4225/49/555C0B8D30C3E	Dryad	http://dx.doi:10.5061/dryad.k9c31
21 Kennington & Hoffmann ^34^ Molecular markers & In(2L)t inversion	Clinal	*Drosophila melanogaster*	Microsatellite markers, Alcohol dehydrogenase (Adh) locus and in(2L)t inversion	Genetic markers	Individual categories	Kennington & Hoffmann ^34^	10.1186/1471-2148-13-100	http://adeer.pearg.com/biogs/DR00290b.htm	https://dx.doi.org/10.4225/49/555C0B8D30C3E	Dryad	http://dx.doi:10.5061/dryad.k9c31
22 Kennington et al. ^41^ Molecular markers & In(3R)Payne inversion	Clinal	*Drosophila melanogaster*	Microsatellite markers, Hsr-omega locus and In(3R)Payne inversion	Genetic markers	Individual measurements	Kennington et al.	10.1534/genetics.105.053173	http://adeer.pearg.com/biogs/DR00292b.htm	https://dx.doi.org/10.4225/49/555C0B8D30C3E	Dryad	http://dx.doi:10.5061/dryad.k9c31
23 Knibb et al. ^46^ Inversion frequencies	Clinal	*Drosophila melanogaster*	Inversions	Genetic markers	Population frequencies	Knibb et al. ^46^	17249108	http://adeer.pearg.com/biogs/DR00296b.htm	https://dx.doi.org/10.4225/49/555C0B8D30C3E	Dryad	http://dx.doi:10.5061/dryad.k9c31
24 Kriesner et al. ^43^ Wolbachia infection frequencies & mtDNA haplotypes	Clinal	*Drosophila simulans*	mtDNA haplotype and wolbachia infection	Genetic markers and endosymbionts	Population counts	Kriesner et al. ^43^	10.1371/journal.ppat.1003607	http://adeer.pearg.com/biogs/DR00065b.htm	https://dx.doi.org/10.4225/49/555C0B8D30C3E	Dryad	http://dx.doi:10.5061/dryad.k9c31
25 Lee et al. ^42^ Neurofibromin gene & In(3R)Payne inversion	Clinal	*Drosophila melanogaster*	Neurofibromin (Nf1) locus and In(3R)Payne inversion	Genetic markers	Individual categories	Lee et al. ^42^	10.1111/mec.12301	http://adeer.pearg.com/biogs/DR00247b.htm	https://dx.doi.org/10.4225/49/555C0B8D30C3E	Dryad	http://dx.doi:10.5061/dryad.k9c31
26 Mitrovski & Hoffmann ^13^ Mean overwinter egg counts and longevity	Clinal	*Drosophila melanogaster*	Overwinter longevity and fecundity	Life history	Subgroup means	Mitrovski & Hoffmann ^13^	10.1098/rspb.2001.1787	http://adeer.pearg.com/biogs/DR00229b.htm	https://dx.doi.org/10.4225/49/555C0B8D30C3E	Dryad	http://dx.doi:10.5061/dryad.k9c31
27 Mitrovski & Hoffmann ^13^ Overwinter temperature, egg laying and mortality rates	Clinal	*Drosophila melanogaster*	Overwinter mortality and fecundity	Life history	Subgroup means	Mitrovski & Hoffmann ^13^	10.1098/rspb.2001.1787	http://adeer.pearg.com/biogs/DR00228b.htm	https://dx.doi.org/10.4225/49/555C0B8D30C3E	Dryad	http://dx.doi:10.5061/dryad.k9c31
28 Mitrovski & Hoffmann ^13^ Overwinter raw egg counts	Clinal	*Drosophila melanogaster*	Overwinter egg count	Life history	Subgroup measurements	Mitrovski & Hoffmann ^13^	10.1098/rspb.2001.1787	http://adeer.pearg.com/biogs/DR00233b.htm	https://dx.doi.org/10.4225/49/555C0B8D30C3E	Dryad	http://dx.doi:10.5061/dryad.k9c31
29 Mitrovski & Hoffmann ^13^ Overwinter raw mortality	Clinal	*Drosophila melanogaster*	Overwinter mortality	Life history	Subgroup measurements	Mitrovski & Hoffmann ^13^	10.1098/rspb.2001.1787	http://adeer.pearg.com/biogs/DR00232b.htm	https://dx.doi.org/10.4225/49/555C0B8D30C3E	Dryad	http://dx.doi:10.5061/dryad.k9c31
30 Oakeshott et al. ^36^ Adh & Gpdh loci	Clinal	*Drosophila melanogaster*	Alcohol dehydrogenase (Adh) and Glycerol-3-phosphate dehydrogenase (Gpdh)	Genetic markers	Population frequencies	Oakeshott et al. ^36^	10.2307/2407970	http://adeer.pearg.com/biogs/DR00298b.htm	https://dx.doi.org/10.4225/49/555C0B8D30C3E	Dryad	http://dx.doi:10.5061/dryad.k9c31
31 Oakeshott et al. ^35^ G6pd locus	Clinal	*Drosophila melanogaster*	Glucose-6-phosphate dehydrogenase (G6pd) locus	Genetic markers	Population frequencies	Oakeshott et al. ^35^	10.1038/hdy.1983.7	http://adeer.pearg.com/biogs/DR00303b.htm	https://dx.doi.org/10.4225/49/555C0B8D30C3E	Dryad	http://dx.doi:10.5061/dryad.k9c31
32 Oakeshott et al. ^35^ Pgd locus	Clinal	*Drosophila melanogaster*	6-phosphogluconate dehydrogenase (Pgd) locus	Genetic markers	Population frequencies	Oakeshott et al. ^35^	10.1038/hdy.1983.7	http://adeer.pearg.com/biogs/DR00304b.htm	https://dx.doi.org/10.4225/49/555C0B8D30C3E	Dryad	http://dx.doi:10.5061/dryad.k9c31
33 Rako et al. ^15^ Post winter male fertility 2006	Clinal	*Drosophila melanogaster*	Post winter male fertility	Life history	Individual measurements	Rako et al. ^15^	10.1111/j.1420-9101.2009.01852.x	http://adeer.pearg.com/biogs/DR00237b.htm	https://dx.doi.org/10.4225/49/555C0B8D30C3E	Dryad	http://dx.doi:10.5061/dryad.k9c31
34 Rako et al. ^15^ Post winter male fertility 2008	Clinal	*Drosophila melanogaster*	Post winter male fertility	Life history	Individual measurements	Rako et al. ^15^	10.1111/j.1420-9101.2009.01852.x	http://adeer.pearg.com/biogs/DR00308b.htm	https://dx.doi.org/10.4225/49/555C0B8D30C3E	Dryad	http://dx.doi:10.5061/dryad.k9c31
35 Rako et al. ^15^ Post winter male size 2006	Clinal	*Drosophila melanogaster*	Post winter wing centroid size	Morphology	Individual measurements	Rako et al. ^15^	10.1111/j.1420-9101.2009.01852.x	http://adeer.pearg.com/biogs/DR00307b.htm	https://dx.doi.org/10.4225/49/555C0B8D30C3E	Dryad	http://dx.doi:10.5061/dryad.k9c31
36 Rako et al. ^15^ Male size 2008	Clinal	*Drosophila melanogaster*	Thorax length and wing centroid size	Morphology	Individual measurements	Rako et al. ^15^	10.1111/j.1420-9101.2009.01852.x	http://adeer.pearg.com/biogs/DR00309b.htm	https://dx.doi.org/10.4225/49/555C0B8D30C3E	Dryad	http://dx.doi:10.5061/dryad.k9c31
37 Telonis Scott et al. ^25^ Raw pcr data	Clinal	*Drosophila melanogaster*	Ebony expression	Genetic markers	Subgroup measurements	Telonis-Scott et al. ^25^	10.1111/j.1365-294X.2011.05089.x	http://adeer.pearg.com/biogs/DR00069b.htm	https://dx.doi.org/10.4225/49/555C0B8D30C3E	Dryad	http://dx.doi:10.5061/dryad.k9c31
38 Telonis Scott et al. ^25^ Raw pigmentation data	Clinal	*Drosophila melanogaster*	Thoracic trident pigmentation scores	Morphology	Individual measurements	Telonis-Scott et al. ^25^	10.1111/j.1365-294X.2011.05089.x	http://adeer.pearg.com/biogs/DR00068b.htm	https://dx.doi.org/10.4225/49/555C0B8D30C3E	Dryad	http://dx.doi:10.5061/dryad.k9c31
39 Telonis Scott et al. ^25^ Average pcr pigmentation	Clinal	*Drosophila melanogaster*	Ebony expression and Thoracic trident pigmentation scores	Genetic markers	Population means	Telonis-Scott et al. ^25^	10.1111/j.1365-294X.2011.05089.x	http://adeer.pearg.com/biogs/DR00070b.htm	https://dx.doi.org/10.4225/49/555C0B8D30C3E	Dryad	http://dx.doi:10.5061/dryad.k9c31
40 Umina et al. ^8^ Adh locus	Clinal	*Drosophila melanogaster*	Alcohol dehydrogenase (Adh) locus	Genetic markers	Population frequencies	Umina et al. ^8^	10.1126/science.1109523	http://adeer.pearg.com/biogs/DR00235b.htm	https://dx.doi.org/10.4225/49/555C0B8D30C3E	Dryad	http://dx.doi:10.5061/dryad.k9c31
41 Umina et al. ^8^ In(3R)Payne inversion	Clinal	*Drosophila melanogaster*	In(3R)Payne inversion	Genetic markers	Population frequencies	Umina et al. ^8^	10.1126/science.1109523	http://adeer.pearg.com/biogs/DR00236b.htm	https://dx.doi.org/10.4225/49/555C0B8D30C3E	Dryad	http://dx.doi:10.5061/dryad.k9c31
42 Van Heerwaarden & Sgro ^16^ D.melanogaster thorax length	Clinal	*Drosophila melanogaster*	Thorax length	Morphology	Individual measurements	Van Heerwaarden & Sgro ^16^	10.1111/j.1558-5646.2010.01196.x	http://adeer.pearg.com/biogs/DR00240b.htm	https://dx.doi.org/10.4225/49/555C0B8D30C3E	Dryad	http://dx.doi:10.5061/dryad.k9c31
43 Van Heerwaarden & Sgro ^16^ D.melanogaster wing centroid size	Clinal	*Drosophila melanogaster*	Wing centroid size	Morphology	Individual measurements	Van Heerwaarden & Sgro ^16^	10.1111/j.1558-5646.2010.01196.x	http://adeer.pearg.com/biogs/DR00239b.htm	https://dx.doi.org/10.4225/49/555C0B8D30C3E	Dryad	http://dx.doi:10.5061/dryad.k9c31
44 Van Heerwaarden & Sgro ^16^ D.melanogaster wing thorax ratio	Clinal	*Drosophila melanogaster*	Wing to thorax ratio	Morphology	Individual measurements	Van Heerwaarden & Sgro ^16^	10.1111/j.1558-5646.2010.01196.x	http://adeer.pearg.com/biogs/DR00241b.htm	https://dx.doi.org/10.4225/49/555C0B8D30C3E	Dryad	http://dx.doi:10.5061/dryad.k9c31
45 Van Heerwaarden & Sgro ^16^ D.simulans thorax length	Clinal	*Drosophila simulans*	Thorax length	Morphology	Individual measurements	Van Heerwaarden & Sgro ^16^	10.1111/j.1558-5646.2010.01196.x	http://adeer.pearg.com/biogs/DR00243b.htm	https://dx.doi.org/10.4225/49/555C0B8D30C3E	Dryad	http://dx.doi:10.5061/dryad.k9c31
46 Van Heerwaarden & Sgro ^16^ D.simulans wing centroid size	Clinal	*Drosophila simulans*	Wing centroid size	Morphology	Individual measurements	Van Heerwaarden & Sgro ^16^	10.1111/j.1558-5646.2010.01196.x	http://adeer.pearg.com/biogs/DR00242b.htm	https://dx.doi.org/10.4225/49/555C0B8D30C3E	Dryad	http://dx.doi:10.5061/dryad.k9c31
47 Van Heerwaarden & Sgro ^16^ D.simulans wing thorax ratio	Clinal	*Drosophila simulans*	Wing to thorax ratio	Morphology	Individual measurements	Van Heerwaarden & Sgro ^16^	10.1111/j.1558-5646.2010.01196.x	http://adeer.pearg.com/biogs/DR00244b.htm	https://dx.doi.org/10.4225/49/555C0B8D30C3E	Dryad	http://dx.doi:10.5061/dryad.k9c31
48 Weeks et al. 2005 Clock locus	Clinal	*Drosophila melanogaster*	Clock locus	Genetic markers	Population frequencies	Weeks et al. 2005	10.1111/j.1420-9101.2005.01013.x	http://adeer.pearg.com/biogs/DR00313b.htm	https://dx.doi.org/10.4225/49/555C0B8D30C3E	Dryad	http://dx.doi:10.5061/dryad.k9c31
49 Weeks et al. 2005 Period locus	Clinal	*Drosophila melanogaster*	Period locus	Genetic markers	Population frequencies	Weeks et al. 2005	10.1111/j.1420-9101.2005.01013.x	http://adeer.pearg.com/biogs/DR00311b.htm	https://dx.doi.org/10.4225/49/555C0B8D30C3E	Dryad	http://dx.doi:10.5061/dryad.k9c31
50 Weeks et al. 2005 ThrGly locus	Clinal	*Drosophila melanogaster*	Clock locus	Genetic markers	Population means	Weeks et al. 2005	10.1111/j.1420-9101.2005.01013.x	http://adeer.pearg.com/biogs/DR00312b.htm	https://dx.doi.org/10.4225/49/555C0B8D30C3E	Dryad	http://dx.doi:10.5061/dryad.k9c31
51 Collinge et al. Cold tolerance	Clinal	*Drosophila melanogaster*	Cold recovery time	Stress resistance	Individual measurements	Collinge et al. ^21^	10.1111/j.1420-9101.2005.01016.x	http://adeer.pearg.com/biogs/DR00320b.htm	https://dx.doi.org/10.4225/49/555C0B8D30C3E	Dryad	http://dx.doi:10.5061/dryad.k9c31
52 Collinge et al. ^21^ Heat tolerance	Clinal	*Drosophila melanogaster*	Heat knockdown time	Stress resistance	Individual measurements	Collinge et al. ^21^	10.1111/j.1420-9101.2005.01016.x	http://adeer.pearg.com/biogs/DR00321b.htm	https://dx.doi.org/10.4225/49/555C0B8D30C3E	Dryad	http://dx.doi:10.5061/dryad.k9c31
53 Collinge et al. ^21^ Ovariole number	Clinal	*Drosophila melanogaster*	Ovariole number	Life history	Individual measurements	Collinge et al. ^21^	10.1111/j.1420-9101.2005.01016.x	http://adeer.pearg.com/biogs/DR00322b.htm	https://dx.doi.org/10.4225/49/555C0B8D30C3E	Dryad	http://dx.doi:10.5061/dryad.k9c31
54 Collinge et al. ^21^ Development time	Clinal	*Drosophila melanogaster*	Development time	Life history	Subgroup means	Collinge et al. ^21^	10.1111/j.1420-9101.2005.01016.x	http://adeer.pearg.com/biogs/DR00323b.htm	https://dx.doi.org/10.4225/49/555C0B8D30C3E	Dryad	http://dx.doi:10.5061/dryad.k9c31
55 Collinge et al. ^21^ Wing area	Clinal	*Drosophila melanogaster*	Wing area	Morphology	Individual measurements	Collinge et al. ^21^	10.1111/j.1420-9101.2005.01016.x	http://adeer.pearg.com/biogs/DR00324b.htm	https://dx.doi.org/10.4225/49/555C0B8D30C3E	Dryad	http://dx.doi:10.5061/dryad.k9c31
56 Collinge et al. ^21^ Egg viability	Clinal	*Drosophila melanogaster*	Egg viability	Life history	Subgroup measurements	Collinge et al. ^21^	10.1111/j.1420-9101.2005.01016.x	http://adeer.pearg.com/biogs/DR00325b.htm	https://dx.doi.org/10.4225/49/555C0B8D30C3E	Dryad	http://dx.doi:10.5061/dryad.k9c31
57 Collinge et al. ^21^ Genetic markers summary	Clinal	*Drosophila melanogaster*	AC008193, DMTRXIII, DMU25686, Hsp70, Hsr-omega	Genetic markers	Population frequencies	Collinge et al. ^21^	10.1111/j.1420-9101.2005.01016.x	http://adeer.pearg.com/biogs/DR00326b.htm	https://dx.doi.org/10.4225/49/555C0B8D30C3E	Dryad	http://dx.doi:10.5061/dryad.k9c31
58 Collinge et al. ^21^ Hsp70 locus	Clinal	*Drosophila melanogaster*	Hsp70 locus	Genetic markers	Population frequencies	Collinge et al. ^21^	10.1111/j.1420-9101.2005.01016.x	http://adeer.pearg.com/biogs/DR00327b.htm	https://dx.doi.org/10.4225/49/555C0B8D30C3E	Dryad	http://dx.doi:10.5061/dryad.k9c31
59 Collinge et al. ^21^ DMTRXIII locus	Clinal	*Drosophila melanogaster*	DMTRXIII locus	Genetic markers	Population frequencies	Collinge et al. ^21^	10.1111/j.1420-9101.2005.01016.x	http://adeer.pearg.com/biogs/DR00328b.htm	https://dx.doi.org/10.4225/49/555C0B8D30C3E	Dryad	http://dx.doi:10.5061/dryad.k9c31
60 Collinge et al. ^21^ Hsr-omega locus	Clinal	*Drosophila melanogaster*	Hsr-omega locus	Genetic markers	Population frequencies	Collinge et al. ^21^	10.1111/j.1420-9101.2005.01016.x	http://adeer.pearg.com/biogs/DR00329b.htm	https://dx.doi.org/10.4225/49/555C0B8D30C3E	Dryad	http://dx.doi:10.5061/dryad.k9c31
61 Collinge et al. ^21^ DMU25686 locus	Clinal	*Drosophila melanogaster*	DMU25686 locus	Genetic markers	Population frequencies	Collinge et al. ^21^	10.1111/j.1420-9101.2005.01016.x	http://adeer.pearg.com/biogs/DR00330b.htm	https://dx.doi.org/10.4225/49/555C0B8D30C3E	Dryad	http://dx.doi:10.5061/dryad.k9c31
62 Collinge et al. ^21^ AC008193 locus	Clinal	*Drosophila melanogaster*	AC008193 locus	Genetic markers	Population frequencies	Collinge et al. ^21^	10.1111/j.1420-9101.2005.01016.x	http://adeer.pearg.com/biogs/DR00331b.htm	https://dx.doi.org/10.4225/49/555C0B8D30C3E	Dryad	http://dx.doi:10.5061/dryad.k9c31
63 McKechnie et al. ^39^ Dca MCA	Clinal	*Drosophila melanogaster*	Drosophila cold acclimation (Dca) locus	Genetic markers	Population frequencies	McKechnie et al. ^39^	10.1111/j.1365-294X.2009.04509.x	http://adeer.pearg.com/biogs/DR00333b.htm	https://dx.doi.org/10.4225/49/555C0B8D30C3E	Dryad	http://dx.doi:10.5061/dryad.k9c31
64 McKechnie et al. ^39^ Dca locus	Clinal	*Drosophila melanogaster*	Drosophila cold acclimation (Dca) locus	Genetic markers	Population counts	McKechnie et al. ^39^	10.1111/j.1365-294X.2009.04509.x	http://adeer.pearg.com/biogs/DR00334b.htm	https://dx.doi.org/10.4225/49/555C0B8D30C3E	Dryad	http://dx.doi:10.5061/dryad.k9c31
65 Lee et al. ^29^ Egg stage 2008	Clinal	*Drosophila melanogaster*	Egg stage	Life history	Individual measurements	Lee et al. ^29^	10.1111/j.1365-294X.2009.04509.x	http://adeer.pearg.com/biogs/DR00335b.htm	https://dx.doi.org/10.4225/49/555C0B8D30C3E	Dryad	http://dx.doi:10.5061/dryad.k9c31
66 Lee et al. ^29^ Egg stage 2009	Clinal	*Drosophila melanogaster*	Egg stage	Life history	Individual measurements	Lee et al. ^29^	10.1111/j.1365-294X.2009.04509.x	http://adeer.pearg.com/biogs/DR00336b.htm	https://dx.doi.org/10.4225/49/555C0B8D30C3E	Dryad	http://dx.doi:10.5061/dryad.k9c31
67 Lee et al. ^29^ Egg stage 2010	Clinal	*Drosophila melanogaster*	Egg stage	Life history	Individual measurements	Lee et al. ^29^	10.1111/j.1365-294X.2009.04509.x	http://adeer.pearg.com/biogs/DR00337b.htm	https://dx.doi.org/10.4225/49/555C0B8D30C3E	Dryad	http://dx.doi:10.5061/dryad.k9c31
68 Lee et al. ^29^ Association analysis	Clinal	*Drosophila melanogaster*	Egg stage and Couch potato (Cpo) locus	Life history and genetic markers	Individual measurements	Lee et al. ^29^	10.1111/j.1365-294X.2009.04509.x	http://adeer.pearg.com/biogs/DR00338b.htm	https://dx.doi.org/10.4225/49/555C0B8D30C3E	Dryad	http://dx.doi:10.5061/dryad.k9c31
69 Lee et al. ^29^ Couch potato locus	Clinal	*Drosophila melanogaster*	Couch potato (Cpo) locus	Genetic markers	Population frequencies	Lee et al. ^29^	10.1111/j.1365-294X.2009.04509.x	http://adeer.pearg.com/biogs/DR00339b.htm	https://dx.doi.org/10.4225/49/555C0B8D30C3E	Dryad	http://dx.doi:10.5061/dryad.k9c31
70 Lee et al. ^29^ Couch potato expression	Clinal	*Drosophila melanogaster*	Couch potato (Cpo) locus expression	Genetic markers	Population means	Lee et al. ^29^	10.1111/j.1365-294X.2009.04509.x	http://adeer.pearg.com/biogs/DR00340b.htm	https://dx.doi.org/10.4225/49/555C0B8D30C3E	Dryad	http://dx.doi:10.5061/dryad.k9c31
71 James et al. ^12^ Thorax length & wing traits	Clinal	*Drosophila melanogaster*	Thorax length, wing area, wing cell area and wing cell number	Morphology	Population means	James et al. ^12^	9215894	http://adeer.pearg.com/biogs/DR00344b.htm	https://dx.doi.org/10.4225/49/555C0B8D30C3E	Dryad	http://dx.doi:10.5061/dryad.k9c31
72 Magiafoglou et al. ^27^ Development time	Clinal	*Drosophila serrata*	Development time	Life history	Population means	Magiafoglou et al. ^27^	10.1046/j.1420-9101.2002.00439.x	http://adeer.pearg.com/biogs/DR00347b.htm	https://dx.doi.org/10.4225/49/555C0B8D30C3E	Dryad	http://dx.doi:10.5061/dryad.k9c31
73 Magiafoglou et al. ^27^ Viability and cold resistance	Clinal	*Drosophila serrata*	Cold resistance and viability	Stress resistance and life history	Population means	Magiafoglou et al. ^27^	10.1046/j.1420-9101.2002.00439.x	http://adeer.pearg.com/biogs/DR00348b.htm	https://dx.doi.org/10.4225/49/555C0B8D30C3E	Dryad	http://dx.doi:10.5061/dryad.k9c31
74 Sgrò et al. ^30^ Longevity	Clinal	*Drosophila melanogaster*	Longevity	Life history	Subgroup means	Sgrò et al. ^30^	10.1111/mec.12353	http://adeer.pearg.com/biogs/DR00255b.htm	https://dx.doi.org/10.4225/49/555C0B8D30C3E	Dryad	http://dx.doi:10.5061/dryad.k9c31
75 Sgrò et al. ^30^ Methuselah expression	Clinal	*Drosophila melanogaster*	Methuselah (mth) locus expression	Genetic markers	Subgroup measurements	Sgrò et al. ^30^	10.1111/mec.12353	http://adeer.pearg.com/biogs/DR00253b.htm	https://dx.doi.org/10.4225/49/555C0B8D30C3E	Dryad	http://dx.doi:10.5061/dryad.k9c31
76 Sgrò et al. ^30^ Methuselah locus	Clinal	*Drosophila melanogaster*	Methuselah (mth) locus	Genetic markers	Individual categories	Sgrò et al. ^30^	10.1111/mec.12353	http://adeer.pearg.com/biogs/DR00254b.htm	https://dx.doi.org/10.4225/49/555C0B8D30C3E	Dryad	http://dx.doi:10.5061/dryad.k9c31
77 Arthur et al. ^28^ Cold resistance	Clinal	*Drosophila simulans*	Cold recovery time	Stress resistance	Individual measurements	Arthur et al. ^28^	10.1111/j.1420-9101.2008.01617.x	http://adeer.pearg.com/biogs/DR00355b.htm	https://dx.doi.org/10.4225/49/555C0B8D30C3E	Dryad	http://dx.doi:10.5061/dryad.k9c31
78 Arthur et al. ^28^ Desiccation resistance	Clinal	*Drosophila simulans*	Desiccation resistance	Stress resistance	Individual measurements	Arthur et al. ^28^	10.1111/j.1420-9101.2008.01617.x	http://adeer.pearg.com/biogs/DR00356b.htm	https://dx.doi.org/10.4225/49/555C0B8D30C3E	Dryad	http://dx.doi:10.5061/dryad.k9c31
79 Arthur et al. ^28^ Development time	Clinal	*Drosophila simulans*	Development time	Life history	Individual measurements	Arthur et al. ^28^	10.1111/j.1420-9101.2008.01617.x	http://adeer.pearg.com/biogs/DR00357b.htm	https://dx.doi.org/10.4225/49/555C0B8D30C3E	Dryad	http://dx.doi:10.5061/dryad.k9c31
80 Loeschcke et al Thorax and wing traits natural pops	Clinal	*Drosophila aldrichi* and *buzzatii*	Thorax length, wing traits	Morphology	Individual measurements	Loeschcke et al. ^18^	10.1046/j.1365-2540.2000.00766.x	http://adeer.pearg.com/biogs/DR00374b.htm	https://dx.doi.org/10.4225/49/555C0B8D30C3E	Dryad	http://dx.doi:10.5061/dryad.k9c31
81 Loeschcke et al Wing traits and asymmetry natural pops	Clinal	*Drosophila aldrichi* and *buzzatii*	Wing traits and wing assymmetry	Morphology	Individual measurements	Loeschcke et al. ^18^	10.1046/j.1365-2540.2000.00766.x	http://adeer.pearg.com/biogs/DR00375b.htm	https://dx.doi.org/10.4225/49/555C0B8D30C3E	Dryad	http://dx.doi:10.5061/dryad.k9c31
82 Loeschcke et al ^18^ Wing asymmetry natural pops	Clinal	*Drosophila aldrichi* and *buzzatii*	Wing assymmetry	Morphology	Individual measurements	Loeschcke et al. ^18^	10.1046/j.1365-2540.2000.00766.x	http://adeer.pearg.com/biogs/DR00376b.htm	https://dx.doi.org/10.4225/49/555C0B8D30C3E	Dryad	http://dx.doi:10.5061/dryad.k9c31
83 Loeschcke et al ^18^ Thorax and wing traits lab pops	Clinal	*Drosophila aldrichi* and *buzzatii*	Thorax length and wing traits	Morphology	Individual measurements	Loeschcke et al. ^18^	10.1046/j.1365-2540.2000.00766.x	http://adeer.pearg.com/biogs/DR00377b.htm	https://dx.doi.org/10.4225/49/555C0B8D30C3E	Dryad	http://dx.doi:10.5061/dryad.k9c31
84 Loeschcke et al ^18^ Wing traits and asymmetry lab pops	Clinal	*Drosophila aldrichi* and *buzzatii*	Wing traits and wing assymmetry	Morphology	Individual measurements	Loeschcke et al. ^18^	10.1046/j.1365-2540.2000.00766.x	http://adeer.pearg.com/biogs/DR00378b.htm	https://dx.doi.org/10.4225/49/555C0B8D30C3E	Dryad	http://dx.doi:10.5061/dryad.k9c31
85 Loeschcke et al ^18^ Wing asymmetry lab pops	Clinal	*Drosophila aldrichi* and *buzzatii*	Assymmetry of wing traits	Morphology	Individual measurements	Loeschcke et al. ^18^	10.1046/j.1365-2540.2000.00766.x	http://adeer.pearg.com/biogs/DR00379b.htm	https://dx.doi.org/10.4225/49/555C0B8D30C3E	Dryad	http://dx.doi:10.5061/dryad.k9c31
86 Loeschcke et al ^18^ Development time & viabiliy lab pops	Clinal	*Drosophila aldrichi* and *buzzatii*	Development time and viability	Life history	Population means	Loeschcke et al. ^18^	10.1046/j.1365-2540.2000.00766.x	http://adeer.pearg.com/biogs/DR00380b.htm	https://dx.doi.org/10.4225/49/555C0B8D30C3E	Dryad	http://dx.doi:10.5061/dryad.k9c31
87 Barker ^37^ Allozyme allele frequencies 67 populations	Clinal	*Drosophila buzzatii*	Allozymes	Genetic markers	Population frequencies	Barker ^37^	10.1111/bij.12067	http://adeer.pearg.com/biogs/DR00361b.htm	https://dx.doi.org/10.4225/49/555C0B8D30C3E	Dryad	http://dx.doi:10.5061/dryad.k9c31
88 Barker ^37^ Allozyme allele frequencies 195 collections	Clinal	*Drosophila buzzatii*	Allozymes	Genetic markers	Population frequencies	Barker ^37^	10.1111/bij.12067	http://adeer.pearg.com/biogs/DR00362b.htm	https://dx.doi.org/10.4225/49/555C0B8D30C3E	Dryad	http://dx.doi:10.5061/dryad.k9c31
89 Barker ^37^ GENEPOP allozyme file 195 collections	Clinal	*Drosophila buzzatii*	Allozymes	Genetic markers	Individual measurements	Barker ^37^	10.1111/bij.12067	http://adeer.pearg.com/biogs/DR00363b.htm	https://dx.doi.org/10.4225/49/555C0B8D30C3E	Dryad	http://dx.doi:10.5061/dryad.k9c31
90 Barker ^37^ Overview 67 Populations	Clinal	*Drosophila buzzatii*	Allozymes	Genetic markers	Population frequencies	Barker ^37^	10.1111/bij.12067	http://adeer.pearg.com/biogs/DR00364b.htm	https://dx.doi.org/10.4225/49/555C0B8D30C3E	Dryad	http://dx.doi:10.5061/dryad.k9c31
91 Barker et al. ^44^ Microsatellite markers	Clinal	*Drosophila buzzatii*	Microsatellite markers	Genetic markers	Individual categories	Barker et al. ^44^	10.1038/hdy.2008.127	http://adeer.pearg.com/biogs/DR00370b.htm	https://dx.doi.org/10.4225/49/555C0B8D30C3E	Dryad	http://dx.doi:10.5061/dryad.k9c31
92 Barker et al. ^44^ GENEPOP microsatellite file	Clinal	*Drosophila buzzatii*	Microsatellite markers	Genetic markers	Individual categories	Barker et al. ^44^	10.1038/hdy.2008.127	http://adeer.pearg.com/biogs/DR00371b.htm	https://dx.doi.org/10.4225/49/555C0B8D30C3E	Dryad	http://dx.doi:10.5061/dryad.k9c31
93 Barker et al. ^44^ Microsatellite allele frequencies	Clinal	*Drosophila buzzatii*	Microsatellite markers	Genetic markers	Population means	Barker et al. ^44^	10.1038/hdy.2008.127	http://adeer.pearg.com/biogs/DR00372b.htm	https://dx.doi.org/10.4225/49/555C0B8D30C3E	Dryad	http://dx.doi:10.5061/dryad.k9c31
94 Barker et al. ^6^ S. aclinata microsatellite markers	Clinal	*Scaptodrosophila aclinata*	Microsatellite markers	Genetic markers	Individual categories	Barker et al. ^6^	10.1038/sj.hdy.6800592	http://adeer.pearg.com/biogs/DR00367b.htm	https://dx.doi.org/10.4225/49/555C0B8D30C3E	Dryad	http://dx.doi:10.5061/dryad.k9c31
95 Barker et al. ^6^ S. hibisci microsatellite markers	Clinal	*Scaptodrosophila hibisci*	Microsatellite markers	Genetic markers	Individual categories	Barker et al. ^6^	10.1038/sj.hdy.6800592	http://adeer.pearg.com/biogs/DR00368b.htm	https://dx.doi.org/10.4225/49/555C0B8D30C3E	Dryad	http://dx.doi:10.5061/dryad.k9c31
96 Magiafoglou et al. ^27^ microsatellite markers	Clinal	*Drosophila serrata*	Microsatellite markers	Genetic markers	Individual categories	Magiafoglou et al. ^27^	10.1046/j.1420-9101.2002.00439.x	http://adeer.pearg.com/biogs/DR00397b.htm	https://dx.doi.org/10.4225/49/555C0B8D30C3E	Dryad	http://dx.doi:10.5061/dryad.k9c31
97 Hoffmann et al. ^32^ Desiccation resistance	Clinal	*Drosophila melanogaster*	Desiccation resistance	Stress resistance	Subgroup measurements	Hoffmann et al. ^32^	10.1111/j.1365-2435.2005.00959.x	http://adeer.pearg.com/biogs/DR00394b.htm	https://dx.doi.org/10.4225/49/555C0B8D30C3E	Dryad	http://dx.doi:10.5061/dryad.k9c31
98 Hoffmann et al. ^32^ Starvation resistance	Clinal	*Drosophila melanogaster*	Starvation resistance	Stress resistance	Subgroup measurements	Hoffmann et al. ^32^	10.1111/j.1365-2435.2005.00959.x	http://adeer.pearg.com/biogs/DR00395b.htm	https://dx.doi.org/10.4225/49/555C0B8D30C3E	Dryad	http://dx.doi:10.5061/dryad.k9c31
99 Hoffmann et al. ^32^ Heat resistance	Clinal	*Drosophila melanogaster*	Heat knockdown time	Stress resistance	Individual measurements	Hoffmann et al. ^32^	10.1111/j.1365-2435.2005.00959.x	http://adeer.pearg.com/biogs/DR00396b.htm	https://dx.doi.org/10.4225/49/555C0B8D30C3E	Dryad	http://dx.doi:10.5061/dryad.k9c31
201 Barker ^5^ S. aclinata collection records	Species distribution	*Scaptodrosophila aclinata*			Presence records	Barker ^5^	10.1038/sj.hdy.6800592	http://adeer.pearg.com/biogs/DR00390b.htm	https://dx.doi.org/10.4225/49/555C0B8D30C3E	Dryad	http://dx.doi:10.5061/dryad.k9c31
202 Barker ^5^ S. hibisci collection records	Species distribution	*Scaptodrosophila hibisci*			Presence records	Barker ^5^	10.1038/sj.hdy.6800592	http://adeer.pearg.com/biogs/DR00391b.htm	https://dx.doi.org/10.4225/49/555C0B8D30C3E	Dryad	http://dx.doi:10.5061/dryad.k9c31
203 Barker et al. ^6^ D. buzzatii and aldrichi collection records	Species distribution	*Drosophila buzzatii* and *D. aldrichi*			Presence records	Barker et al. ^6^	10.1111/j.1442-9993.2005.01470.x	http://adeer.pearg.com/biogs/DR00387b.htm	https://dx.doi.org/10.4225/49/555C0B8D30C3E	Dryad	http://dx.doi:10.5061/dryad.k9c31
204 Schiffer & McEvey ^7^ Montium colletion records	Species distribution	*Drosophila bunnanda, D. serrata, D. birchii, D. kikkawai* and *D.* sp. cf. *jambulina*			Presence records	Schiffer & McEvey ^7^	na	http://adeer.pearg.com/biogs/DR00342b.htm	https://dx.doi.org/10.4225/49/555C0B8D30C3E	Dryad	http://dx.doi:10.5061/dryad.k9c31
Collection, species and traits studied, data type, the original publication, data depository, data record accession, DOI for ADEER and Dryad, and DOI (or PMID) for the original publication are listed for each dataset.											

**Table 2:** Phenotypic traits and genetic markers studied along the eastern Australian coast in drosophilid species

**Trait/genetic marker**	**Species**	**Clinal variation**	**Clinal pattern**	**References**
**Morphological**				
* *Egg size	*D. melanogaster*	Yes	Linear	Azevedo et al. ^20^
* *Pigmentation	*D. melanogaster*	Yes	Linear	Telonis Scott et al. ^25^
* *Thorax length	*D. melanogaster*	Yes	Linear	James et al. ^17^, ^12^, Rako et al. ^15^
	*D. melanogaster*	No		Van Heerwaarden & Sgrò ^16^
	*D. melanogaster*	Na		Hoffmann et al. ^10^
	*D. aldrichi*	Na		Loeschcke et al. ^18^
	*D. buzzatii*	Na		Loeschcke et al. ^18^
	*D. simulans*	Yes	Linear	Van Heerwaarden & Sgro ^16^
* *Mass	*D. serrata*	Yes	Linear and quadratic	Hallas et al. ^19^
* *Wing size	*D. melanogaster*	Yes	Linear	Gockel et al. ^22^, James et al. ^17^, ^12^, Collinge et al. ^21^, Rako et al. ^15^, Van Heerwaarden & Sgrò ^16^
	*D. aldrichi*	Na		Loeschcke et al. ^18^
	*D. buzzatii*	Na		Loeschcke et al. ^18^
	*D. birchii*	No		Griffiths et al. ^23^
	*D. serrata*	Yes	Linear and quadratic	Hoffmann & Shirriffs ^24^, Hallas et al. ^19^
	*D. simulans*	Yes	Linear	Van Heerwaarden & Sgro ^16^
* *Wing to aspect ratio	*D. melanogaster*	Yes, in the field	Linear	Azevedo et al. ^11^
* *Wing to thorax ratio	*D. melanogaster*	Yes	Linear	Azevedo et al. ^11^, Van Heerwaarden & Sgrò ^16^
	*D. aldrichi*	Na		Loeschcke et al. ^18^
	*D. buzzatii*	Na		Loeschcke et al. ^18^
	*D. simulans*	No		Van Heerwaarden & Sgro ^16^
* *Wing shape	*D. aldrichi*	Na		Loeschcke et al. ^18^
	*D. buzzatii*	Na		Loeschcke et al. ^18^
* *Fluctuating asymmetry	*D. buzzatii*	Na		Loeschcke et al. ^18^
	*D. aldrichi*	Na		Loeschcke et al. ^18^
* *Lipid content	*D. melanogaster*	Na		Hoffmann et al. ^10^
				
**Life-history**				
* *Development time	*D. melanogaster*	Yes	Linear	James & Partridge ^26^, Collinge et al. ^21^
	*D. aldrichi*	Na		Loeschcke et al. ^18^
	*D. buzzatii*	Na		Loeschcke et al. ^18^
	*D. birchii*	Yes	Linear	Griffiths et al. ^23^
	*D. serrata*	Yes	Linear and quadratic	Magiafoglou et al. ^27^
	*D. simulans*	No		Arthur et al. ^28^
* *Longevity	*D. melanogaster*	Yes	Linear and quadratic	Sgrò et al. ^30^
* *Overwinter mortality	*D. melanogaster*	Yes	Linear and quadratic	Mitrovski & Hoffmann ^13^, Hoffmann et al. ^14^
* *Overwinter fecundity	*D. melanogaster*	Yes	Linear and quadratic	Mitrovski & Hoffmann ^13^
	*D. melanogaster*		Linear	Hoffmann et al. ^14^, Rako et al. ^15^
* *Mortality	*D. aldrichi*	Na		Loeschcke et al. ^18^
	*D. buzzatii*	Na		Loeschcke et al. ^18^
	*D. serrata*	Yes	Linear	Magiafoglou et al. ^27^
* *Timing of overwinter fecundity	*D. melanogaster*	Yes	Linear	Mitrovski & Hoffmann ^13^, Hoffmann et al. ^14^
* *Ovariole number	*D. melanogaster*	Yes	Quadratic	Azevedo et al. ^20^
	*D. melanogaster*		Linear	Collinge et al. ^21^
* *Ovarian dormancy	*D. melanogaster*	Yes	Quadratic	Lee et al. ^29^
				
**Stress**				
* *Cold resistance	*D. melanogaster*	Yes	Linear	Hoffmann et al. ^10^, ^31^, Collinge et al. ^21^
	*D. melanogaster*	Na		Hoffmann et al. ^10^
	*D. birchii*	No		Griffiths et al. ^23^
	*D. serrata*	Yes	Linear	Hallas et al. ^19^, Magiafoglou et al. ^27^
	*D. simulans*	Yes, females	Cubic	Arthur et al. ^28^
* *Desiccation resistance	*D. melanogaster*	No		Hoffmann et al. ^10^, Hoffmann et al. ^32^
	*D. melanogaster*	Na		Hoffmann et al. ^10^
	*D. birchii*	Yes	Linear	Griffiths et al. ^23^
	*D. serrata*	No		Hallas et al. ^19^
	*D. simulans*	No		Arthur et al. ^28^
* *Heat resistance	*D. melanogaster*	Yes	Linear	Hoffmann et al. ^31^, Collinge et al. ^21^, Hoffmann et al. ^10^
	*D. birchii*	No		Griffiths et al. ^23^
* *Starvation resistance	*D. melanogaster*	Yes, females	Linear	Hoffmann et al. ^10^
	*D. melanogaster*	No		Hoffmann et al. ^32^
	*D. birchii*	Yes	Linear	Griffiths et al. ^23^
	*D. serrata*	Yes, males	Linear	Hallas et al. ^19^
				
**Inversions**				
* *In(2L)t	*D. melanogaster*	Yes	Linear	Knibb et al. ^46^
	*D. melanogaster*	Na		Kennington & Hoffmann ^34^
* *In(2R)NS	*D. melanogaster*	Yes	Linear	Knibb et al. ^46^
* *In(3L)Payne	*D. melanogaster*	Yes	Linear	Knibb et al. ^46^
* *In(3R)Payne	*D. melanogaster*	Yes	Linear	Knibb et al. ^46^, Lee et al. ^42^, Umina et al. ^8^, Kennington et al ^41^
* *In(3R)C	*D. melanogaster*	Yes	Linear	Knibb et al. ^46^
				
**Allozymes**				
* *Adh	*D. melanogaster*	Yes	Linear	Oakeshott et al. ^36^, Umina et al. ^8^
	*D. melanogaster*	Na		Kennington & Hoffmann ^34^
* *Gpdh	*D. melanogaster*	Yes	Linear	Oakeshott et al. ^36^
* *G6pd	*D. melanogaster*	Yes	Linear	Oakeshott et al. ^35^
* *Pgd	*D. melanogaster*	Yes	Linear	Oakeshott et al. ^35^
* *Pgm, Aldox, Hex, Adh, Est1, Est2 and Lap	*D. buzzatii*	Yes	Linear	Barker ^37^
				
**DNA sequence variation**				
* clock*	*D. melanogaster*	No		Weeks et al. 2005
* couch potato*	*D. melanogaster*	Yes	Linear	Lee et al. ^29^
* drosophila cold acclimation*	*D. melanogaster*	Yes	Linear	Mckechnie et al. ^39^
* frost*	*D. melanogaster*	Yes	Linear	Hoffmann et al. ^38^
* hsp70*	*D. melanogaster*	No		Collinge et al. ^21^
* hsr-omega*	*D. melanogaster*	Yes	Linear	Kennington et al. ^41^, Collinge et al. ^21^
* methuselah*	*D. melanogaster*	Yes	Linear and quadratic	Sgrò et al. ^30^
* neurofibromin*	*D. melanogaster*	Yes	Linear	Lee et al. ^42^
* period*	*D. melanogaster*	No		Weeks et al. 2005
* *MtDNA	*D. simulans*	Na		Kriesner et al. ^43^
				
**DNA repeat variation**				
* *Microsatellite markers	*D. melanogaster*	Yes, 5 out of 19	Linear	Gockel et al. ^22^
	*D. melanogaster*	Yes, 9 out of 24	Linear	Kennington et al. ^41^
	*D. melanogaster*	Na		Kennington & Hoffmann ^34^
	*D. buzzatii*	Yes, 6 out of 15	Linear	Barker 2009
	*D. serrata*	No		Magiafoglou et al. ^27^
	*S. aclinata*	Na		Barker et al. ^6^
	*S. hibisci*	Na		Barker et al. ^6^
				
**Gene expression**				
* *couch potato	*D. melanogaster*	Yes	Linear and quadratic	Lee et al. ^29^
* *ebony	*D. melanogaster*	Yes, at 25 °C	Linear	Telonis-Scott et al. ^25^
* *methuselah	*D. melanogaster*	Yes	Linear	Sgrò et al. ^30^
				
**Others**				
* Wolbachia*	*D. simulans*	Na		Kriesner et al. ^43^
The presence and pattern of clinal variation and the publication associated with the datasets are reported				

**Table 3:** Species distribution datasets for nine drosophilid species

**Datasets**	**Species**	**Publication**	**Collection years**
201 Barker^5^ S. aclinata collection records	*S. aclinata*	Barker^5^	1995
202 Barker^5^ S. hibisci collection records	*S. hibisci*	Barker^5^	1998
203 Barker *et al*.^6^ D. buzzatii and aldrichi collection records	*D. buzzatii*	Barker *et al*.^6^	1971-2002
203 Barker *et al*.^6^ D. buzzatii and aldrichi collection records	*D. aldrichi*	Barker *et al*.^6^	1971-2002
204 Schiffer & McEvey^7^ Montium collection records	*D. bunnanda*	Schiffer & McEvey^7^	1924-2005
204 Schiffer & McEvey^7^ Montium collection records	*D. serrata*	Schiffer & McEvey^7^	1924-2005
204 Schiffer & McEvey^7^ Montium collection records	*D. birchii*	Schiffer & McEvey^7^	1924-2005
204 Schiffer & McEvey^7^ Montium collection records	*D. kikkawai*	Schiffer & McEvey^7^	1924-2005
204 Schiffer & McEvey^7^ Montium collection records	*D.* sp. cf. *jambulina*	Schiffer & McEvey^7^	1924-2005
The publication associated with the dataset and collection years are reported.			
